# On the role of STAT1 and STAT6 ADP-ribosylation in the regulation of macrophage activation

**DOI:** 10.1038/s41467-018-04522-z

**Published:** 2018-06-01

**Authors:** Andreas Begitt, James Cavey, Mathias Droescher, Uwe Vinkemeier

**Affiliations:** Division of Infections, Immunity and Microbes, School of Life Sciences, University of Nottingham, Nottingham, NG7 2UH UK

## Introduction

The post translational modification of transcription factors is an important regulatory mechanism in gene expression. Well-studied examples are the transcription factors STAT1 and STAT6, which are tyrosine phosphorylated (“activated”) in response to interferon-γ (IFN-γ) and interleukin-4 (IL-4), respectively. In their recent paper in *Nature Communications* Iwata et al. report that STAT1 and STAT6 are targets of another post-translational modification, namely mono ADP-ribosylation, and that this modification controls their tyrosine phosphorylation^1^. This is potentially a highly relevant observation, since the activated STATs play antagonistic roles in controlling the activity and polarization of macrophages. Iwata et al. therefore conclude that modification of STAT1 and STAT6 by ADP-ribosylation is critical for the cross-regulation of macrophages by IFN-γ and IL-4 and the development of atherosclerosis. However, this interpretation is called into question by the authors’ failure to consider another previously described post-translational modification namely the SUMO conjugation of STAT1, which is a critical regulator of IFN-γ signaling and macrophage activity^2^.

**Fig. 1:**
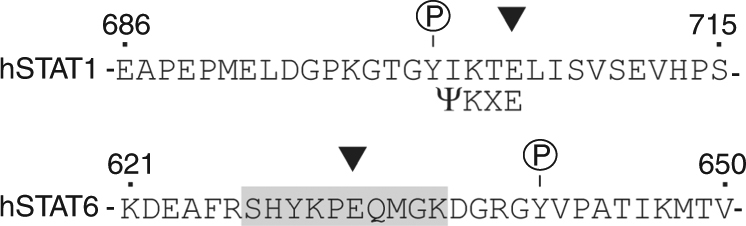
The purported ADP-ribosylation site of STAT1 is not conserved for STAT6 and overlaps with the SUMO consensus sequence. Amino acid sequences flanking the phosphorylation site of human STAT1 (top) and human STAT6 (bottom). The phosphorylated tyrosyl residues are indicated by P (in circles). Proposed ADP-ribosylation sites are indicated by black triangles. The canonical SUMO consensus sequence ψ-K-X-E (ψ, a hydrophobic amino acid; K, SUMO acceptor, X, any amino acid residue) is given underneath the respective STAT1 sequence. The proposed ADP-ribosylated STAT6 peptide is highlighted by a gray rectangle

Iwata et al. identify the ADP-ribosyltransferases PARP9 and PARP14 as potential regulators of macrophage activation through proteomics screenings and data clustering. Their subsequent biochemical experiments indicate that PARP14 suppresses gene induction by IFN-γ and augments IL-4 responses, whereas PARP9 promotes IFN-γ–induced gene activities. They observe that PARP14 silencing increased the phosphorylation of STAT1 upon IFN-γ stimulation and decreased the phosphorylation of STAT6 upon IL4 stimulation in a human monocytic cell line. While these results provide a rationale for the concurrent changes in cytokine-induced transcription, they do not link this phenomenon to ADP ribosylation of transcription factors STAT1 and STAT6. In order to establish such a direct relationship between PARP expression and STAT modification, the authors use purified recombinant proteins for enzymatic assays followed by mass spectrometry, which identified Glu657 and Glu705 as sites of STAT1 ADP-ribosylation in vitro. Expression of STAT1 mutated at both residues to Gln to preclude modification indeed demonstrates elevated IFN-γ-induced tyrosine phosphorylation and accordingly increased expression of pro-inflammatory STAT1 target genes. This outcome is in line with the authors’ observation that silencing of the ADP-ribosyltransferase PARP14 is associated with the same phenotype, and the authors conclude that STAT1 is ADP-ribosylated in living cells despite the absence of data demonstrating this. What is more, the congruence of reduced PARP expression and increased STAT1 phosphorylation is taken as indication that ADP-ribosylation somehow interferes with the tyrosine phosphorylation of STAT1. The authors point out the proximity of putative ADP-ribosylation sites (residue 657 and 705) and the phosphorylated tyrosine 701, but do not provide experimental evidence or in fact a molecular explanation for how this proximity might diminish STAT1 activity. Crucially, the authors do not acknowledge that the alleged ADP-ribosylation site forms a vital part of the consensus sequence for another post-translational modification, namely the well-documented conjugation of STAT1 with SUMO at Lys703 (Fig. 1)^3, 4^. Several mutations including Glu705 to Gln used by Iwata et al. have previously been described that inactivate the SUMO consensus sequence and preclude STAT1 sumoylation^5, 6^. As SUMO conjugation and tyrosine phosphorylation of STAT1 are mutually exclusive, the lack of SUMO conjugation leads to the phenotype described by Iwata et al., i.e., elevated Tyr701 phosphorylation, increased expression of pro-inflammatory STAT1 target genes, and clinical disease^6–8^. We note in passing though that atherosclerosis and vascular disease, which Iwata et al. link to reduced STAT1 ADP-ribosylation, do not appear to be among the severe ailments that affect patients with a mutation at the alleged STAT1 ADP-ribosylation site Glu705^9^. The STAT1 gain-of-function phenotype caused by the mutation of Glu705, which Iwata et al. describe in their work as a novel finding, thus merely confirms previously published results. Of note, the mutational approach cannot dissociate the effects of SUMOylation and purported ADP ribosylation on STAT1. It is therefore important to consider that silencing of the SUMO-conjugating enzyme Ubc9, which precludes specifically the SUMO conjugation of STAT1, likewise results in enhanced STAT1 tyrosine phosphorylation^6^. This, in turn, indicates that lack of ADP-ribosylation is unlikely to result in the STAT1-gain-of-function associated with mutation of the purported ADP-ribosylation site, Glu705. Whilst any involvement of ADP-ribosylation cannot be completely ruled out, the phenotype, including increased macrophage activation in response to IFN-γ, can be explained by the absence of SUMO conjugation alone.

In addition, the authors implicate ADP-ribosylation of another STAT protein, STAT6, in the regulation of macrophages. Using purified STAT6 in the same in vitro enzyme assay described for STAT1 followed by mass spectrometry, Iwata et al. identify a ribosylated peptide but could not verify the modified residue. For STAT1, the ADP-ribosyl-conjugated glutamic acid residue is in position +4 of the functionally critical Tyr701 phosphorylation site. Since for STAT6 the ADP-ribosylated peptide likewise harbors a Glu residue in close proximity (+3) to tyrosine residue 629 that according to the authors is phosphorylated and the functional equivalent of STAT1 Tyr701, the authors infer from its conserved positioning that Glu632 is a plausible candidate for the STAT6 ADP-ribosylation site (Fig. 1). However, this needs explanation, as for considerable time now the field agrees on Tyr641 as the functionally critical and possibly sole phosphorylated tyrosine of human STAT6^10^. In contrast, to-date and to the best of our knowledge phosphorylation at Tyr629 has not been demonstrated including by Iwata et al. Thus, the presumed ADP-ribosylation sites of STAT6 and STAT1 bear little if any conservation, which undermines the authors’ proposal of shared regulatory mechanisms for the IFN-γ-STAT1 and IL-4-STAT6 pathways by PARP9 and PARP14.

In conclusion, based on bioinformatic and proteomic analyses the work of Iwata et al. suggests that the ADP-ribosyltransferases PARP9 and PARP14 contribute to the regulation of macrophage activity and polarization. Our comments do not exclude this possibility. However, in light of knowledge that the authors failed to consider in their interpretations, their central claim that this process is dependent on the ADP-ribosylation of STAT1 and STAT6 is unfounded at present.

### Data availability

No datasets were generated during the current study.
